# Characteristics of patients with primary open angle glaucoma and normal tension glaucoma at a university hospital: a cross-sectional retrospective study

**DOI:** 10.1186/s13104-015-1339-x

**Published:** 2015-08-19

**Authors:** Yu Yokoyama, Kazuichi Maruyama, Hideyuki Konno, Sayaka Hashimoto, Mai Takahashi, Hiroko Kayaba, Taiki Kokubun, Toru Nakazawa

**Affiliations:** Department of Ophthalmology, Tohoku University Graduate School of Medicine, 1-1, Seiryo-machi, Aoba-ku, Sendai, Miyagi 980-8574 Japan; Department of Retinal Disease Control, Tohoku University Graduate School of Medicine, Miyagi, Japan; Department of Advanced Ophthalmic Medicine, Tohoku University Graduate School of Medicine, Miyagi, Japan

**Keywords:** Glaucoma, Visual field, Progression of visual field defects, Intraocular pressure

## Abstract

**Background:**

The characteristics of glaucoma patients and their response to therapy may differ by institution, region and country. Therefore, clinicians should understand the distinctiveness of their patients. Here, we profile primary open angle glaucoma (POAG) and normal tension glaucoma (NTG) patients at a major university hospital in Japan.

**Methods:**

This study included 523 eyes from 523 POAG and NTG patients who underwent full clinical ophthalmologic evaluations at Tohoku University Hospital. Clinical characteristics such as age, sex, visual acuity, intraocular pressure, Humphrey field analyzer-measured mean deviation (MD) and MD slope were collected retrospectively. MD slope was calculated from MD data that included the first baseline measurement of MD and 4 subsequent, consecutive, reliable measurements of MD. Refractive error was analyzed in a subgroup with no history of refractive surgery, including intraocular lens implantation. Patient characteristics were analyzed separately in the groups of patients with low (<15 mmHg) and high IOP (≥15 mmHg) and in the groups with MD slope ≥−1.0 and <−1.0 dB/year.

**Results:**

Mean age, visual acuity (median), IOP, pre-treatment IOP (from patient history), refractive error and MD were 61.7 ± 12.5 years, −0.08 (interquartile range −0.08 to 0.05) LogMAR, 13.87 ± 3.37 mmHg, 18.35 ± 6.26 mmHg, −4.48 ± 3.81 diopters and −11.73 ± 8.83 dB, respectively. POAG and NTG patients had significant differences in mean age (63.4 ± 12.4 vs. 60.7 ± 12.5 years, *P* < 0.01), visual acuity, IOP (14.95 ± 4.20 vs. 13.21 ± 2.54 mmHg, *P* < 0.01) and MD (−13.85 ± 9.32 vs. −10.45 ± 8.27 dB, *P* < 0.01). Interestingly, MD slope was slightly steeper in the low-IOP group than in the high-IOP group, although the difference was not statistically significant (−0.85 vs. −0.70 dB/year, *P* = 0.31). Baseline MD was significantly worse in the group with MD slope <−1.0 dB/year than in the group with MD slope ≥−1.0 dB/year (−11.56 vs. −7.64 dB/year, *P* < 0.01).

**Conclusions:**

We identified characteristics of glaucoma patients at a university hospital that may reflect the specialized nature of such an institution.

## Background

Data from population-based studies show that the number of glaucoma patients over 40 years old now exceeds 60 million worldwide, making glaucoma the second leading cause of blindness. Furthermore, these data indicate that the number of patients will reach 80 million by 2020 [1]. Treatment for glaucoma patients currently relies on the maintenance of low intraocular pressure (IOP), which has been shown to be an effective method of preventing glaucoma progression [2–4]. However, recent investigations have shown that glaucoma is a multifactorial disease, and that the complicated nature of its causes and progression can affect treatment efficacy. Therefore, decisions on treatment for patients with progressive glaucoma must take into consideration the background of the patient and the type of glaucoma [5–9].

Recent knowledge on glaucoma chiefly comes from a number of multicenter studies that had large and diverse study populations, chosen to represent the circumstances of ordinary clinical practice. However, patient populations varied between these previous studies in distribution of glaucoma type, especially primary open angle glaucoma (POAG) and normal tension glaucoma (NTG), and in the distribution of characteristics such as age, progression speed and glaucoma severity. Thus, it can be unclear for clinicians whether the evidence from these studies is applicable to any particular clinic. There is therefore a need for more sources of information on the profiles of glaucoma patients at a variety of institutions, in order to better understand patient characteristics and possible differences with reported research findings.

Past studies have not provided details on how patient populations may vary between different types of institutions. Thus, the present cross-sectional study sought to clarify the characteristics of POAG and NTG patients at a central institution: a central hospital whose specific role is to provide care for community medicine.

## Subjects and methods

This study was retrospective and cross-sectional. All experimental procedures were conducted in accordance with the tenets set forth in the Declaration of Helsinki. This study was approved by the Institutional Review Board of the Tohoku University Graduate School of Medicine (2014-1-56). All data had previously been retained by the research-implementing entity and were anonymized. Research not involving human biological specimens, such as the present study, does not necessarily require informed consent from the subjects, according to the Ethical Guidelines for Medical and Health Research Involving Human Subjects. Furthermore, we made information on our research available on the homepage of our institution, including the purpose of our information-gathering activities. We also provided, on the homepage, an opportunity for potential research subjects to refuse to be involved, or to discontinue involvement.

A total of 523 eyes of 523 patients with POAG or NTG were recruited from the ophthalmology outpatient clinic of Tohoku University Hospital. This institution is located in Sendai, the largest city in northeastern Japan, and is the central hospital of Miyagi Prefecture. All patients were treated by glaucoma specialists who followed the Japan Glaucoma Society Guidelines for Glaucoma [10]. Data were also collected from a list of 1812 glaucoma patients who underwent full clinical ophthalmologic evaluations at the ophthalmology outpatient clinic of Tohoku University Hospital. These ophthalmologic evaluations included testing for visual acuity, refractive error and IOP with Goldmann applanation tonometry, as well as slit lamp and fundus examinations. Standard automated perimetry was performed with the Humphrey field analyzer (HFA; SITA standard 24-2 or 30-2). Patients with eye diseases or a history of ophthalmic surgeries affecting the visual field were excluded.

Best-corrected visual acuity was measured with a decimal visual acuity chart and converted into LogMAR units. The spherical equivalent, determined as spherical power plus half the cylindrical power, was used to represent refractive error. Refractive error was analyzed in a subgroup of patients that had no history of refractive surgery, including intraocular lens implantation. The analysis of visual field loss included only reliable data with rates of fixation loss <33 %, false positives <15 %, and false negatives <15 %.

Open angle glaucoma was diagnosed in this study based on: (1) the presence of morphological change in the optic nerve head, such as excavation of the cup and notching of the rim, (2) thinning of the retinal nerve fiber layer (RNFL), determined by OCT scanning, (3) visual field defects corresponding to damaged areas of the RNFL and (4) a wide, open anterior chamber angle. Open angle glaucoma was diagnosed as POAG if the pre-treatment IOP was more than 21 mmHg, and as NTG if IOP was 21 mmHg or less.

Visual fields were assessed with the HFA. Glaucomatous visual fields were defined by the presence of at least one of the following: (1) the results of a glaucoma hemifield test were outside the normal limits; (2) a cluster of three or more non-edge points was present at a location typical for glaucoma, with all points being depressed on the pattern deviation plot to a *P* < 5 % level and at least one point depressed to a *P* < 1 % level; and (3) significant corrected pattern standard deviation at the *P* < 5 % level.

The latest cross-sectional data available from the glaucoma patients were collected retrospectively. A final total of 523 eyes of 523 patients were included in the study, from the originally recruited 1812 patients. The data were excluded when an eye with glaucoma could not be diagnosed as having either POAG or NTG, if the eyes had unreliable mean deviation (MD) data due to factors such as fixation loss, or if there was a history of other diseases or surgery affecting the visual field. If neither eye met the exclusion criteria, one was randomly selected for the analysis. Finally, 138 eligible eyes were further selected from the 523 eyes for an MD trend analysis. The MD slope was calculated from five HFA measurements, including the first baseline measurement of MD and 4 subsequent, consecutive, reliable measurements of MD. No eyes included in the MD trend analysis underwent surgery during the study period. Clinical parameters were compared in the groups of patients with low (<15 mmHg) and high IOP (≥15 mmHg), and in the groups with MD slope <−1.0 and ≥−1.0 dB/year.

### Statistical analysis

Statistical analysis was performed with JMP pro 10.02 for Windows (SAS Institute Inc.). Continuous variables were expressed as the mean ± standard deviation, or as the median (interquartile range). The Mann–Whitney U test was used to investigate differences in continuous values for clinical characteristics between the groups, while Fisher’s exact test was used to analyze categorical values. A linear regression analysis was used to investigate the relationship between IOP and MD slope.

## Results

**Fig. 1:**
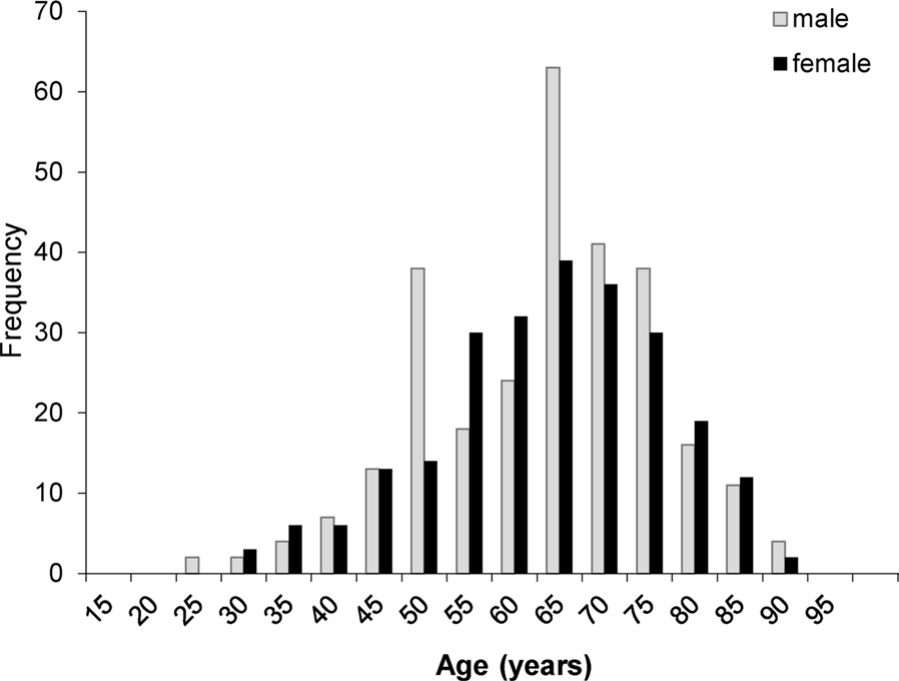
Histogram of subjects

**Fig. 2:**
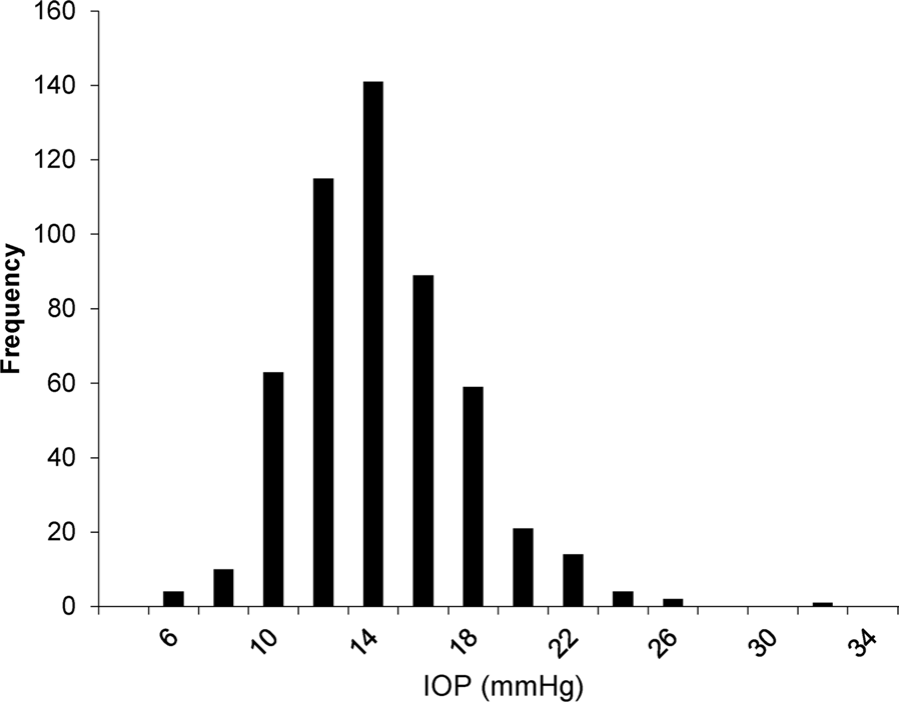
Histogram of intraocular pressure. *IOP* intraocular pressure

**Fig. 3:**
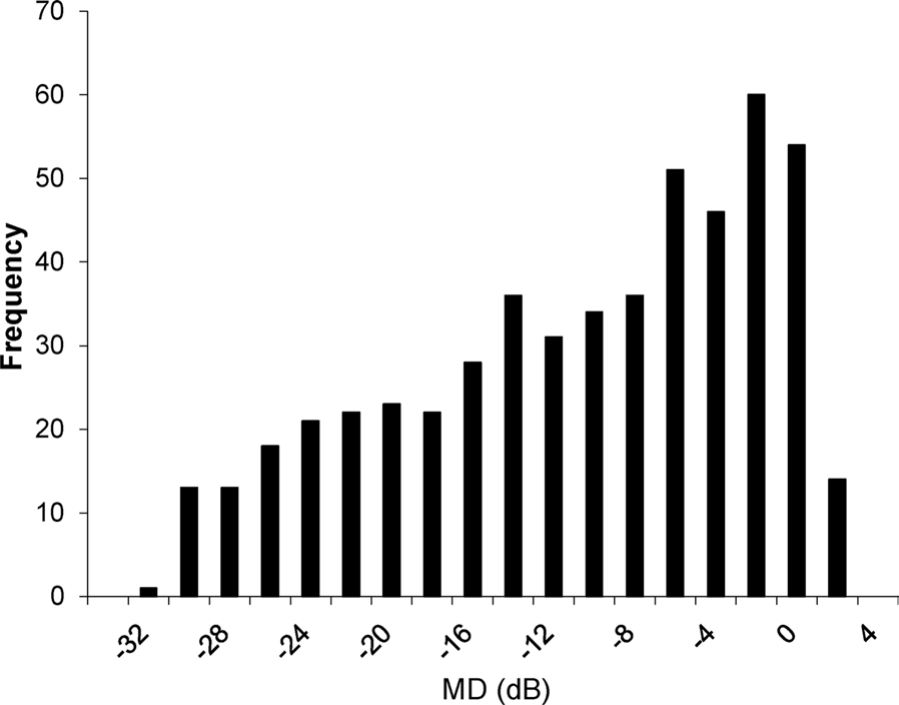
Histogram of mean deviation. *MD* mean deviation

The characteristics of the 523 eyes of 523 patients with POAG or NTG included in this study are shown in Table 1. The patients had a mean age of 61.7 ± 12.5 years (ranging from 21 to 87 years; distribution shown in Fig. 1). The sex ratio was 281:242 (male:female). Median visual acuity (LogMAR), IOP, HFA MD, and HFA pattern standard deviation were −0.08 (interquartile range −0.08 to 0.05), 13.87 ± 3.37 mmHg, 11.73 ± 8.83 and 8.84 ± 4.24 dB, respectively (Table 1). The peak of the IOP distribution curve was shifted downwards despite the presence of glaucoma in the patients, reflecting their use of IOP-lowering treatments (Fig. 2). Our data for the distribution of MD was even, (Fig. 3), reflecting the high percentage (41 %) of patients in our study with severe visual field damage (<−12 dB). There were differences between the POAG and NTG patients in age, sex, visual acuity, IOP and MD (Table 1). The POAG patients had significantly higher IOP than the NTG patients (14.95 vs. 13.21 mmHg). The POAG patients also had more advanced visual field deterioration than the NTG patients (−13.85 vs. −10.38 dB). A trend analysis of MD in 138 eligible eyes revealed that average MD slope was −0.77 dB/year (Table 2). In these patients, mean IOP and mean MD were 14.30 ± 3.44 mmHg and −10.82 ± 7.96 dB. In these eyes, the mean pre-treatment IOP (obtained from the patients’ clinical history) was 18.35 ± 6.26 mmHg.

**Table 1:** Demographics of glaucoma patients

Clinical characteristics	All (N = 523)	POAG (N = 198)	NTG (N = 325)	*P* value (POAG vs. NTG)
Age (years)	61.7 ± 12.5	63.4 ± 12.4	60.7 ± 12.5	<0.01*
Sex (male:female)	281:242	127:71	154:171	<0.01*
VA (LogMAR)	−0.08 (−0.08 to 0.05)^a^	0.00 (−0.08 to 0.15)^a^	−0.08 (−0.08 to 0.00)^a^	<0.01*
IOP (mmHg)	13.87 ± 3.37	14.95 ± 4.20	13.21 ± 2.54	<0.01*
Refractive error (D)^b^	−4.48 ± 3.81 (N = 361)	−4.07 ± 3.68 (N = 122)	−4.69 ± 3.86 (N = 239)	0.15
MD (dB)	−11.73 ± 8.83	−13.85 ± 9.32	−10.45 ± 8.27	<0.01*
PSD (dB)	8.84 ± 4.24	9.02 ± 4.13	8.73 ± 4.31	0.42

Mean ± standard deviation

*VA* visual acuity, *IOP* intraocular pressure, *MD* mean deviation, *PSD* pattern standard deviation, *POAG* primary open-angle glaucoma, *NTG* normal-tension glaucoma

* Significance at *P* < 0.05

^a^Median (interquartile range)

^b^Data from eyes without refractive surgery

**Table 2:** Characteristics of patients with IOP ≥15 or <15 mmHg

Clinical characteristics	All (N = 138)	IOP ≥15 mmHg (N = 69)	IOP <15 mmHg (N = 69)	*P* value (≥15 vs. <15 mmHg)
Age (years)	60.5 ± 11.9	59.9 ± 11.8	61.1 ± 12.1	0.60
Sex (male:female)	72:66	39:30	33:36	0.39
POAG:NTG	46:92	32:37	14:55	<0.01*
VA (LogMAR)	−0.08 (−0.08 to 0.00)^a^	−0.08 (−0.08 to 0.00)^a^	−0.08 (−0.08 to 0.05)^a^	0.12
Refractive error (D)^b^	−4.46 ± 3.64 (N = 124)	−4.23 ± 3.47 (N = 65)	−4.72 ± 3.82 (N = 59)	0.61
IOP (mmHg)	14.30 ± 3.44	17.12 ± 2.34	11.49 ± 1.54	–
MD (dB)	−10.82 ± 7.96	−8.91 ± 7.29	−12.73 ± 8.19	<0.01*
Baseline MD (dB)	−9.23 ± 7.59	−7.31 ± 7.04	−11.15 ± 7.68	<0.01*
MD slope (dB/year)	−0.77 ± 0.98	−0.70 ± 0.95	−0.85 ± 1.02	0.31
Observation period (month)	28.4 ± 14.0	32.8 ± 16.0	23.9 ± 9.9	<0.01*

Mean ± standard deviation

*VA* visual acuity, *IOP* intraocular pressure, *MD* mean deviation, *PSD* pattern standard deviation, *POAG* primary open-angle glaucoma, *NTG* normal-tension glaucoma, *D* diopter

* Significance at *P* < 0.05

^a^Median (interquartile range)

^b^Data from eyes without refractive surgery

**Fig. 4:**
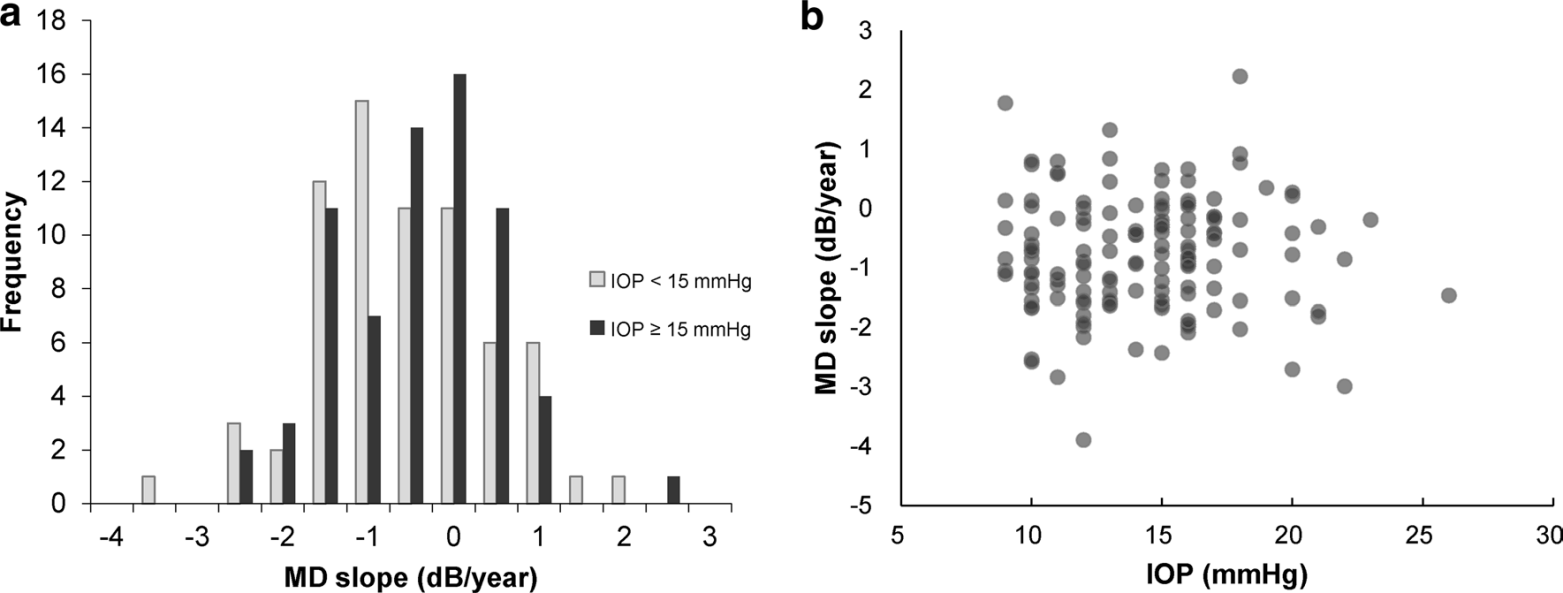
MD slopes in the low-IOP and high-IOP groups. **a** Histogram of MD slope in each group. Even with IOP <15 mmHg, 48 % of patients had progressive visual field defects with MD slope <−1.0 dB/year. In addition, 26 % of these patients had MD slope <−1.5 dB/year. **b** Scatter plot of IOP and MD slope. There was no significant correlation between treated IOP and MD slope (R^2^ = 0.00, *P* = 0.93)

Clinical characteristics were analyzed in the low-IOP (<15 mmHg) and high-IOP (≥15 mmHg) groups (Table 2). This analysis revealed that the eyes with low IOP had more severe glaucomatous damage, with a relatively steeper MD slope, although the difference was not statistically significant (*P* value = 0.31). Furthermore, an analysis of clinical characteristics in the groups with MD slope <−1.0 and ≥−1.0 dB/year groups (Table 3) revealed significant differences in visual acuity, MD, and baseline MD between the two groups. A histogram of the MD slope is shown in Fig. 4a. Among our patients, visual field deterioration progressed at more than −1.0 dB/year in 48 % of eyes with IOP <15 mmHg. Moreover, we did not observe a correlation between IOP and MD slope (R^2^ = 0.00, *P* = 0.93; Fig. 4b).

**Table 3:** Characteristics of patients with MD slope ≥−1.0 or <−1.0 dB/year

Clinical characteristics	All (N = 138)	MD slope ≥−1.0 dB/year (N = 82)	MD slope <−1.0 dB/year (N = 56)	*P* value (≥−1.0 vs. <−1.0)
Age (years)	60.5 ± 11.9	60.7 ± 12.0	61.1 ± 11.9	0.64
Sex (male:female)	72:66	42:40	30:26	0.71
POAG:NTG	46:92	26:56	20:36	0.86
VA (LogMAR)	−0.08 (−0.08 to 0.00)^a^	−0.08 (−0.18 to 0.00)^a^	−0.04 (−0.08 to 0.05)^a^	<0.01*
Refractive error (D)^b^	−4.46 ± 3.64 (N = 124)	−4.34 ± 3.60 (N = 77)	−4.65 ± 3.72 (N = 47)	0.74
IOP (mmHg)	14.30 ± 3.44	14.59 ± 3.27	13.89 ± 3.67	0.11
MD (dB)	−10.82 ± 7.96	−8.27 ± 7.74	−14.56 ± 6.74	<0.01*
Baseline MD (dB)	−9.23 ± 7.59	−7.64 ± 7.61	−11.56 ± 7.00	<0.01*
MD slope (dB/year)	−0.77 ± 0.98	−0.70 ± 0.95	−1.70 ± 0.56	–
Observation period (month)	28.4 ± 14.0	31.2 ± 16.0	24.2 ± 8.8	0.02*

Mean ± standard deviation

*VA* visual acuity, *IOP* intraocular pressure, *MD* mean deviation, *PSD* pattern standard deviation, *POAG* primary open-angle glaucoma, *NTG* normal-tension glaucoma, *D* diopter

* Significance at *P* < 0.05

^a^Median (interquartile range)

^b^Data from eyes without refractive surgery

## Discussion

In the present study, we investigated the characteristics of POAG and NTG patients at a central hospital located in the Tohoku area of Japan, with a large population of patients. The patients in our institution were relatively old, commonly had myopia and had glaucomatous visual field defects with a mean MD of −11.73 dB. Our investigation also included an examination of the rate of visual field deterioration in the glaucoma patients and its relationship with post-treatment IOP. We found that overall mean MD slope was −0.77 dB/year, while patients with low IOP had a slope of −0.85 dB/year and patients with high IOP had a slope of −0.70 dB/year, with a 22 % IOP reduction compared to pre-treatment IOP in both groups. Additionally, we performed a comparison of the clinical characteristics of the two groups, i.e., those with MD slope <−1.0 dB/year and those ≥−1.0 dB/year. This revealed that glaucoma patients with MD slope <−1.0 dB/year had worse baseline MD.

It is well known that myopia is more common in Asia than in Western Europe, the United States or Australia [11–16]. Furthermore, a number of studies have demonstrated that myopia has an impact on the occurrence of glaucoma [17–19]. The Blue Mountains Eye Study demonstrated a relationship between glaucoma and low myopia (between −1.0 and −3.0 diopters), finding that the odds ratio (OR) for glaucoma in eyes with low myopia was 2.3, after adjusting for known glaucoma risk factors [17]. That study also found that eyes with moderate to high myopia had a higher risk (OR: 3.3). Moreover, the Tajimi Study in Japan showed that the OR for glaucoma of eyes with moderate to high myopia (greater than −3.0 D) was 2.6 [19]. The patients in this study had a mean refractive error of −4.48 D, indicating that myopia was common. This may be due to our hospital being located in Japan, a country with a relatively high number of cases of POAG and NTG that are associated with myopia, or may be due to myopic patients with glaucoma being more often referred to central hospitals for specialized treatment.

The Tajimi Study also obtained the information on disease types, revealing that NTG was more common than POAG in Japan [20]. Our study confirmed this finding, although our study population had a higher ratio of POAG to NTG patients. This difference may be due our study being conducted at a large, central institution, with a greater focus on specialized care. Our study population therefore included many patients with relatively severe glaucoma, uncontrolled ocular hypertension or requiring surgical treatment. Additionally, we found that mean IOP in our POAG patients was low, 14.95 mmHg, and very low in the NTG patients, 13.21 mmHg. The value for both groups together was 13.87 mmHg, in contrast to the Tajimi study, which showed that mean IOP in a normal Japanese population, without glaucoma, was 14.5 mmHg. The relatively low IOP in our patients indicates that ocular hypertension was controlled by the glaucoma treatment they received, despite the cross-sectional nature of our IOP data and the likely inclusion of data from patients undergoing changes in their treatment regime, at a stage when IOP was still relatively uncontrolled.

Overall, our results showed that while treatment to reduce IOP could stop or slow the progression of visual field defects in some glaucoma patients, other patients continued to show progression (Fig. 4a). The mean MD slope in the low- and high-IOP groups was −0.85 and −0.70 dB/year, respectively. Surprisingly, we found that 48 and 26 % of low-IOP patients had MD slopes steeper than −1.0 and −1.5 dB/year, respectively. This result disagrees with previous studies, which showed that IOP control in patients with ocular hypertension or glaucoma was an effective treatment, and could prevent or slow the occurrence and progression of glaucoma [2, 4, 21]. Moreover, previous studies found that glaucoma patients with successfully controlled IOP had a mean MD slope, determined with the HFA, of −0.36 to −0.78 dB/year [22–24]. One of these studies, the Advanced Glaucoma Intervention Study (AGIS), confirmed the effectiveness of IOP reduction as a glaucoma treatment by showing that a group of patients with IOP <14 mmHg had a lower incidence of visual field deterioration than a group with IOP <17.5 mmHg. By contrast, the patients in our study with relatively low and high IOP had statistically similar outcomes. In fact, the low-IOP group in our study showed a slightly steeper MD slope than the high-IOP group.

There are two possible reasons that our patients had a relatively high progression rate of visual field deterioration despite successfully reduced IOP. The first reason lies in the multifactorial nature of glaucoma [5–9], now widely recognized, although high IOP is still the most widely known major risk factor. In the Collaborative Normal Tension Glaucoma Study (CNTGS), approximately 20 % of NTG patients had progressive visual field deterioration despite IOP reductions of more than 30 % [25]. The CNTGS thus elucidated not only the effects of IOP reduction on glaucoma but also the limitations of such treatment, and showed that factors besides high IOP have an impact on the pathogenesis and progression of glaucoma. Indeed, previous studies showed that many clinical characteristics associated with glaucoma, such as disc hemorrhage, initial advanced visual field defects, an enlarged vertical cup-to-disc ratio and β-zone peripapillary atrophy (PPA), were related to continued deterioration of the visual field [26–29], in addition to high IOP. Therefore, the relatively high incidence of older patients with more severe glaucoma at our institution might have been caused by IOP-independent progression of visual field defects. In this study, glaucoma patients with an MD slope <−1.0 dB/year had worse baseline visual field defects. This finding agrees with past studies and reflects the limitations of IOP reduction as a treatment for advanced glaucoma.

A second explanation for our observation of relatively fast glaucoma progression, even with treatment, is that our data were collected from an institution serving a disproportionate number of severe cases. The glaucoma patients in this study were referred to a central hospital, treated with such methods as filtering surgery, and then transferred back to the referring hospital if their visual field loss was successfully halted. As a result, the characteristics of the patients at such a central hospital may vary slightly from those in general population studies, and may have included a relatively high number of patients with progressive glaucoma that resisted treatment. This effect is perhaps most strongly evident in our finding that MD slope in a low IOP group was steeper than in a high IOP group, although the difference was not significant. These patients underwent continued aggressive IOP-lowering treatment despite its reduced effectiveness in their cases, because lowering IOP is still the only evidence-based treatment for glaucoma. This would also explain our finding that the low IOP group included more NTG patients (80 %) and more patients with advanced visual field defects than the high IOP group.

### Limitations

The present study was cross-sectional and retrospective. The data were obtained from patients that visited a large, central hospital for different lengths of treatment and underwent therapy with different goals. The data from this cross-sectional, retrospective study may therefore be of somewhat limited use in performing trend analyses, such as for MD slope. Additionally, we were unable to collect baseline and overall IOP data for the entire study period because the glaucoma patients had complicated clinical histories. For example, baseline IOP is usually collected with a non-contact tonometer when patients initially visit a primary clinic. This makes it difficult to collect baseline IOP data obtained with Goldmann tonometry. Finally, we did not investigate risk factors for glaucoma progression such as disc hemorrhage or β-zone PPA. A prospective study would enable us to take such risk factors into account, and to analyze their relationship to damage caused by glaucoma.

Although the data in this study were collected retrospectively, a sufficient number of patients were included to provide us with good information on the distribution of characteristics of glaucoma patients. The aim of the present study was to profile the characteristics of glaucoma patients at a large university hospital, and to emphasize the importance of investigating the specific characteristics of patients in individual institutions. We believe we were able to achieve this goal.

## Conclusions

We investigated the distribution of POAG and NTG patients in a large, central hospital in Japan. At this institution, patients with glaucoma and relatively high myopia were common, as were patients with advanced glaucoma. Although the data in this study were obtained retrospectively, we found that a significant number of patients with well-controlled IOP still showed progression. This finding indicates that the profile of glaucoma patients in different institutions may vary depending on specific institutional characteristics. Thus, every institution should investigate the characteristics of the patients it serves, in order to most accurately apply the findings of past studies.

## Abbreviations

IOP: intraocular pressure
POAG: primary open angle glaucoma
NTG: normal tension glaucoma
HFA: humphrey field analyzer
MD: mean deviation
RNFL: retinal nerve fiber layer
OR: odds ratio
AGIS: Advanced Glaucoma Intervention Study
CNTGS: Collaborative Normal Tension Glaucoma Study
PPA: peripapillary atrophy
